# High fever after sublingual administration of misoprostol for treatment of post‐partum haemorrhage: a hospital‐based, prospective observational study in Argentina

**DOI:** 10.1111/tmi.13389

**Published:** 2020-04-16

**Authors:** Jill Durocher, Jesus Daniel Aguirre, Ilana G. Dzuba, Elba Mirta Morales, Guillermo Carroli, Jesica Esquivel, Roxanne Martin, Cecilia Berecoechea, Beverly Winikoff

**Affiliations:** ^1^ Gynuity Health Projects New York NY USA; ^2^ Hospital Materno Neonatal E.T. de Vidal Corrientes Argentina; ^3^ Centro Rosarino de Estudios Perinatales Rosario Argentina; ^4^ Hospital Llano Corrientes Argentina

**Keywords:** misoprostol, fever, post‐partum haemorrhage, sublingual, Argentina, Latin America, misoprostol, fièvre, hémorragie post‐partum, HPP, sublinguale, Argentine, Amérique latine

## Abstract

**Objective:**

To characterise the occurrence of fever (≥38.0°C) after treatment for post‐partum haemorrhage (PPH) with sublingual misoprostol 800 mcg in Latin America, where elevated rates of misoprostol’s thermoregulatory effects and recipients’ increased susceptibility to high fever have been documented.

**Methods:**

A prospective observational study in hospitals in Argentina enrolled consenting women with atonic PPH after vaginal delivery, eligible to receive misoprostol. Corporal temperature was assessed at 30, 60, 90 and 120 min post‐treatment; other effects were recorded. The incidence of high fever ≥ 40.0°C (primary outcome) was compared to the rate observed previously in Ecuador. Logistic regressions were performed to identify clinical and population‐based predictors of misoprostol‐induced fever.

**Results:**

Transient shivering and fever were experienced by 75.5% (37/49) of treated participants and described as acceptable by three‐quarters of women interviewed (35/47). The high fever rate was 12.2% (6/49), [95% Confidence Interval (CI) 4.6, 24.8], compared to Ecuador’s rate following misoprostol treatment (35.6% (58/163) [95% CI 28.3, 43.5], *P* = 0.002). Significant predictors of misoprostol‐induced fever (model dependent) were as follows: pre‐delivery haemoglobin < 11.0g/dl, rapid placental expulsion, and higher age of the woman. No serious outcomes were reported prior to discharge.

**Conclusions:**

Misoprostol to treat PPH in Argentina resulted in a significantly lower rate of high fever than in Ecuador, although both are notably higher than rates seen elsewhere. A greater understanding of misoprostol’s side effects and factors involved in their occurrence, including genetics, will help alleviate concerns. The onset of shivering may be the simplest way to know if fever can also be expected.

## Abstract (fr)

**Objectif:**

Caractériser la survenue de fièvre (≥ 38,0°C) après traitement d'une hémorragie post‐partum (HPP) avec du misoprostol sublingual à 800 mcg en Amérique latine, où des taux élevés d'effets thermorégulateurs du misoprostol et une sensibilité accrue des receveurs à une forte fièvre ont été documentés.

**Méthodes:**

Une étude observationnelle prospective dans des hôpitaux en Argentine a recruté des femmes consentantes atteintes d'HPP atonique après un accouchement vaginal éligibles pour recevoir du misoprostol. La température corporelle a été évaluée 30, 60, 90 et 120 minutes après le traitement; d'autres effets ont été enregistrés. L'incidence d'une fièvre élevée ≥40,0°C (critère principal) a été comparée au taux observé précédemment en Equateur. Des régressions logistiques ont été effectuées pour identifier les prédicteurs cliniques et ceux basés sur la population de la fièvre induite par le misoprostol .

**Résultats:**

Des frissons transitoires et de la fièvre ont été ressentis par 75% (37/49) des participantes traitées et décrits comme acceptables par les trois quarts des femmes interrogées (35/47). Le taux de fièvre élevé était de 12% (6/49), [intervalle de confiance (IC) à 95%: 4,6, 24,8] contre 35,6% en Equateur après traitement au misoprostol (58/163) [IC95%: 28,3, 43,5], *p* = 0,002). Les prédicteurs significatifs de la fièvre induite par le misoprostol (selon le modèle) étaient: hémoglobine avant l'accouchement <11,0 g/dL, expulsion placentaire rapide et âge plus élevé de la femme. Aucun résultat sévère n'a été signalé avant le sortie d’hôpital.

**Conclusions:**

Le misoprostol pour traiter l'HPP en Argentine a entraîné un taux de fièvre élevée significativement plus bas qu'en Equateur, bien que les taux dans les deux pays soient notablement plus élevés que les taux observés ailleurs. Une meilleure compréhension des effets secondaires du misoprostol et des facteurs impliqués dans leur apparition, y compris la génétique, aidera à atténuer les inquiétudes. L'apparition de frissons peut être le moyen le plus simple de savoir si l'on peut également s'attendre à de la fièvre.

## Introduction

Post‐partum haemorrhage (PPH) remains a significant contributor to maternal death and morbidity worldwide, including in Latin America [1, 2]. Poor outcomes attributable to PPH are largely preventable when uterotonic agents are available and used efficiently to control atonic haemorrhage. Oxytocin is the first‐line intervention recommended for managing PPH due to uterine atony, but it requires parenteral administration and an effective cold chain [3, 4, 5, 6, 7, 8]. If oxytocin is not available, PPH treatment guidelines recommend sublingual misoprostol 800 mcg as an effective alternative to oxytocin infusion [3, 4, 5, 6, 7, 8]. Guidelines also specify the possible need to administer misoprostol to treat refractory atonic haemorrhage when bleeding is not controlled by oxytocin [3, 4, 6, 7, 8].

Misoprostol’s pill‐based administration and heat stability make it a versatile treatment option in a range of situations. However, increased pyrexia associated with misoprostol, particularly the possibility for high fever (≥40.0°C /104°F), has evoked concerns among some providers [2, 9, 10, 11, 12], prompting continued vigilance and investigation of misoprostol’s thermoregulatory effects [9]. A review of studies that report on high fever post‐treatment with sublingual misoprostol 800 mcg reveals that this effect is mostly negligible [13, 14, 15, 16, 17], with the exception of a 36% high fever rate documented in Ecuador [18]. This rate is particularly striking when compared to rates ranging from 0 to 9% in the eight sites in four different countries that participated in two large multi‐country trials evaluating the safety and efficacy of 800 mcg sublingual misoprostol for PPH treatment [13, 14, 18]. Over the years, several case reports have also described rare and unexpected occurrences of high fever following post‐partum misoprostol administration [9, 10, 11, 19, 20]. In some cases, high fever was reportedly accompanied by other alarming effects, including delirium and convulsions [10, 11]. These experiences underscore the need to understand high fever and who is at risk of developing it after exposure to misoprostol for PPH management.

Because of Ecuador’s exceptionally high rate of high fever, a genetic pre‐disposition was hypothesised [12, 18, 21, 22] and later confirmed [23], showing a positive association between two prostaglandin transporter genes and misoprostol‐induced fever among Ecuadorian women who received PPH treatment with misoprostol. We, therefore, conducted research to confirm high fever rates following PPH treatment with misoprostol in another Latin American population, Argentina, to improve our understanding of misoprostol’s side effects and predictors of elevated temperature. PPH continues to be an important cause of morbidity and mortality in Argentina [24, 25, 26]. Furthermore, there are relatively high PPH rates in the region [27, 28] and misoprostol is widely available and used in Latin America, as it is included in local norms for PPH treatment [6, 29, 30].

## Methods

An open‐label study utilising misoprostol (Gymiso, Linepharma, Paris, France) for PPH management was conducted at two tertiary‐level, public hospitals (Hospitals Vidal and Llano), in Corrientes, Argentina. The study sought to evaluate the side effect profile and the occurrence of high fever following treatment with sublingual misoprostol 800 mcg for PPH after vaginal birth. The study was registered with Clinicaltrials.gov (NCT02163616) on 13 June 2014 and was approved by an institutional review board (Centro Rosarino de Estudios Perinatales, Argentina on 16 Oct 2013 (reference #2/13)) and the national regulatory body (Administración Nacional de Medicamentos, Alimentos y Tecnología Médica, on 22 May 2014 (reference #147‐7075/14‐4).

From September 2015 to May 2016, women presenting in active labour were screened for eligibility and invited to participate in the study. Women who had a known allergy to a prostaglandin, a planned caesarean‐section, or who were unable to give consent for any reason were ineligible. After written informed consent, women who planned to deliver vaginally were enrolled and baseline data collected. If a woman required transfer to the operating theatre, she was later excluded. Women were eligible to receive the study treatment, which consisted of a total of four 200 mcg tablets of misoprostol placed under the tongue if their bleeding reached 500 ml and was suspected to be due to uterine atony. PPH recognition was facilitated by using a calibrated, polyurethane receptacle (Brasss‐V Drapes^®^, Excellent Fixable Drapes, India) after the baby’s birth to help quantify the amount of blood lost. Socio‐demographic and delivery characteristics, as well as blood loss at 30‐ and 60‐min post‐partum, were documented for all participants. Pulse and blood pressure (BP) were measured at 15‐min intervals during the first hour post‐partum using a standardised automated instrument (Omron BP785, Omron Healthcare Co., Ltd.).

Treatment packets with misoprostol 800 mcg were prepared by the research team, prior to study launch. Upon diagnosis of PPH due to atonic uterus, hospital staff opened the next consecutively numbered study packet and instructed women to maintain the four tablets under their tongues for 15 min before swallowing any remaining pill fragments. Women requiring additional care beyond a therapeutic misoprostol regimen were offered standard treatments available at each hospital, including additional uterotonics (IV oxytocin and/or IM ergometrine) and IV fluids. Removal of placental products under general anaesthesia and provision of plasma expanders and blood transfusion were done as needed. Blood loss measurement continued until cessation of active bleeding. Laparotomy and/or hysterectomy were performed as a last resort. All actions taken to manage the haemorrhage were documented. Time to bleeding cessation, total post‐partum blood loss, the woman’s general condition and her Hb change from pre‐ to post‐delivery (assessed using a standardised hand‐held device (Hemocue®)), were also recorded.

Women receiving misoprostol had their temperature measured at 30, 60, 90 and 120 min post‐treatment and were observed for any other secondary effects related to the medicine. Temperature was assessed for a minimum of 2 h post‐treatment in order to capture the peak temperature following misoprostol administration, which is known to occur 60–90 min after its administration [18, 22]. Digital thermometers were provided to the sites to standardise temperature assessment. If fever was suspected at any time during the woman’s hospital stay, temperature and time were recorded. For any woman with a high fever ≥ 40.0°C, temperature was measured every half‐hour until the fever subsided (measuring below 38.0°C). Fever was managed by applying cool compresses and administering diclofenac (IV) and/or dipyrone (1 ampoule IM or IV) as per standard practices. Providers documented treatments to manage any side effect and rated the severity of the observed side effect as mild, moderate or severe. Prior to hospital discharge, nurses interviewed participants regarding acceptability of the misoprostol treatment and any side effect experienced.

The primary aim of this study was to document the rate of high fever (≥40.0°C) experienced by women after PPH treatment with 800 mcg misoprostol given sublingually. To compare this rate to the previously reported rate of high fever (36%) in Ecuador following treatment with the same regimen [18], we required a minimum sample of 39 PPH cases to achieve a precision level of ±15%, with an alpha of 0.05. The sample was further increased by 10% to 43 to account for loss to follow‐up or missing temperature readings for assessment of the primary outcome.

An online data entry platform was created by investigators from the Centro Rosarino de Estudios Perinatales to capture all collected data. Data were analysed using the Statistical Package for the Social Sciences, version 20.0 (SPSS, Chicago, IL, USA). Descriptive analyses were conducted to summarise background characteristics, PPH outcomes and severity of any side effect experienced among women treated with misoprostol. Fever ≥ 40.0°C at any time was categorised as high, regardless of its duration; temperatures between 38.0 and 39.9°C were considered to be mild/moderate. The percentage of women with high fever was calculated with its exact binomial 95% confidence interval (CI) and compared to the previously reported high fever rate from Ecuador to confirm if statistically different (*P*‐value < 0.05).

To assess for predictors of misoprostol‐induced fever, we ran two sets of logistic regressions using high fever (≥40.0°C) and fever (≥38.0°C) as the dependent variables. The fever threshold of ≥ 38.0°C is commonly used as an alert sign for other complications post‐delivery [31]; thus, a better understanding of predictors of misoprostol‐induced fever, irrespective of peak temperature, may help providers differentiate this drug effect from other conditions. In both models, we included demographic and obstetric factors as predictor variables, which were previously found to be associated with elevated temperature after misoprostol administration, specifically pre‐delivery Hb, duration of the third stage of labour and ethnicity [18, 22]. Other variables were included in the models if they were thought to potentially influence temperature post‐partum or the woman’s febrile response to the medication or if on bivariate analysis they demonstrated a correlation with fever. Odds ratios (ORs) were calculated for each variable in the regression and their associated 95% CIs. All variables with *P*‐values < 0.05 in the final multivariate model were considered significant predictors of fever.

## Results

A total of 635 women consented to participate and were enrolled. Fifty‐nine women underwent caesarean deliveries after enrolment and became ineligible. Another participant was excluded due to a retained placenta necessitating her transfer to the operating theatre for removal. Immediately after vaginal delivery, 575 participants were given oxytocin prophylaxis and had their blood loss measured using the standardised receptacle. Overall, 12.2% (70/575) were diagnosed with PPH based on ≥ 500 ml blood loss and other clinical factors. On average, PPH was diagnosed approximately 30 min after birth of the baby (range 5–68 min). Uterine atony was deemed to be the primary cause of bleeding in 87.1% (61/70) of cases.

Among the 61 women with atonic PPH, 49 were administered treatment with sublingual misoprostol. One woman was erroneously administered intravenous oxytocin and an IM injection of ergometrine instead of misoprostol. At one participating hospital, 11 additional eligible women received standard uterotonic therapies during a temporary stock‐out of the study medicine. All women treated with misoprostol were given four tablets (800 mcg) sublingually, except for one case in which the provider administered only two tablets (400 mcg). Table 1 describes the participant characteristics and delivery outcomes for the women treated with misoprostol.

**Table 1:** Participant characteristics and delivery outcomes for women administered treatment with sublingual misoprostol for PPH (*n* (%) unless otherwise noted)

	*n* = 49
*Background characteristics*	
Age (year), median (range)	21 (16–42)
Education level	
None	2 (4.0)
Primary	26 (53.1)
Secondary or higher	21 (42.9)
Currently married	23 (46.9)
Number of pregnancies (including current), median (range)	2 (1–10)
Gestational age (weeks), median (range)	39.7 (33.7–41.9)
Self‐described ethnicity	
White (i.e. European)	32 (65.3)
Mestiza (i.e. mixed race of indigenous/Spanish decent)	17 (34.7)
Other illness/conditions	
None	46 (93.9)
Gestational hypertension	1 (2.0)
Urinary traction infection	2 (4.1)
Pre‐delivery haemoglobin (g/dl), median (range)	11.8 (9.1–14.7)
Pre‐delivery Hb < 11.0 g/dl	12 (24.5)
Pre‐delivery temperature (Celsius), median (range)	36.4 (34.2–37.5)
*Delivery characteristics*	
Singleton	49 (100)
Epidural given	0 (0)
Labour induced with uterotonics	8 (16.3)
Labour augmented with uterotonics	35 (71.4)
Episiotomy	19 (38.8)
Oxytocin given during third stage of labour	49 (100)
Controlled cord traction	37 (75.5)
Uterine massage	22 (44.9)
Time of birth to placental expulsion (min), median (range)	5 (0–15)
*PPH outcomes*	
Total blood loss, median (range)	1000 (550–1800)
Additional uterotonics	
Oxytocin (IV)*	7 (14.3)
0.2 ergometrine (IM) + oxytocin (IV)*	4 (8.2)
Exploration under general anaesthesia	1 (2.0)
Plasma expanders	2 (4.1)
Blood transfusion	1 (2.0)
Post‐partum Hb, median (range)	(*n* = 45) 9.8 (6.5–15.7)

* The dose of oxytocin IV given ranged between 10 and 40 IU.

The median blood loss at the time of misoprostol treatment administration was 600 ml (IQR 520, 725) and after bleeding cessation it was 1000 ml (IQR 750, 1200). Eleven women received additional uterotonics (Table 1). Other interventions included plasma expanders (*n* = 2), transfusion of 1 unit of whole blood (*n* = 1) and uterine exploration with general anaesthesia (*n* = 1). No hysterectomies or other surgeries were done. One participant who had received misoprostol treatment and additional uterotonics for moderate blood loss (920 ml) and was discharged in good condition two days after delivery but died two months later in her home, likely due to post‐partum cardiomyopathy, a rare condition after childbirth that is frequently fatal.

**Figure 1:**
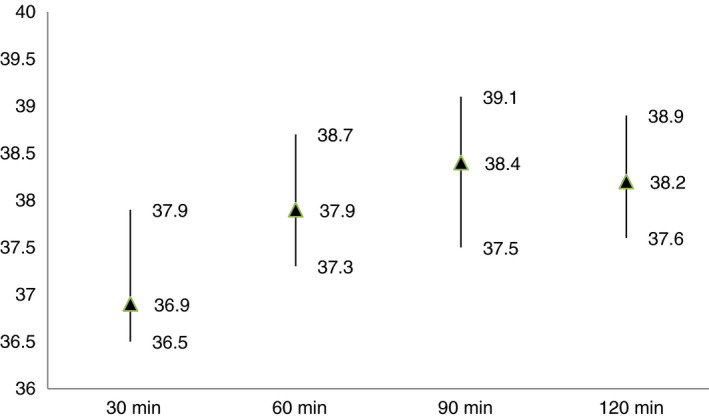
Median temperatures (°C) at 30‐, 60‐, 90‐ and 120‐min post‐treatment with sublingual misoprostol (includes upper/lower quartiles) (*n* = 49).

The majority of women experienced misoprostol’s thermoregulatory effects – shivering (75.5%, 37/49) and fever (75.5%, 37/49) – after taking the medicine; all other side effects were negligible (Table 2). Fever, irrespective of its severity, generally occurred 1–2 h post‐treatment. Average temperatures were notably higher during the second hour after taking misoprostol than the first hour (Figure 1). One‐third of women (11/37) were confirmed to be afebrile (<38.0°C) at the 2‐h post‐treatment temperature assessment. More than half of women with fever were administered a medication to help alleviate symptoms (56.8%; 21/37), which generally consisted of an IV dose of dipyrone. 75% (35/47) of women deemed the overall side effect profile as acceptable (Table 2).

**Table 2:** Other side effects and women’s acceptability of the misoprostol treatment (*n* = 49)

	*n* (%)
*Side effects noted by provider*	
Chills/shivering	
None	12 (24.5)
Mild	20 (40.8)
Moderate	12 (24.5)
Severe	5 (10.2)
Other side effects reported*	
Nausea/vomiting	3 (6.1)
Allergic reaction (rhinitis, epiphora)	1 (2.0)
Itchy palms	1 (2.0)
Women’s acceptability of side effects at the exit interview	(*n* = 47)
Acceptable	35 (74.5)
Neutral	9 (19.1)
Unacceptable	2 (4.3)†
Don’t know	1 (2.1)

* All other side effects were described as either mild or moderate.

† Both women had experienced high fever, one of whom also complained of itchy palms.

**Figure 2:**
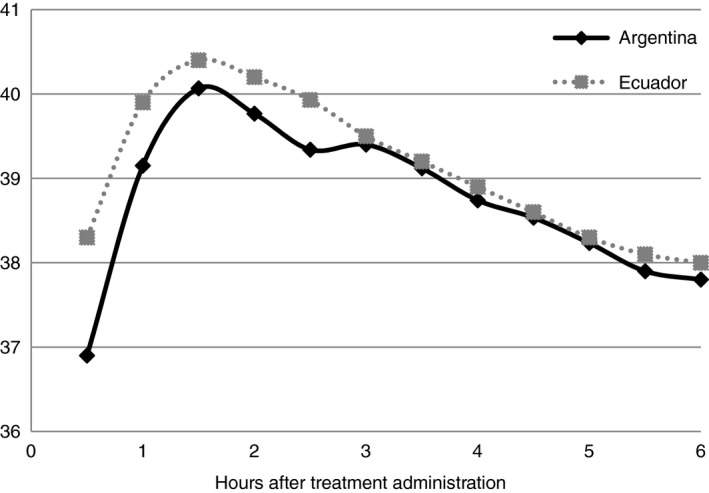
Median temperatures (°C) over time for cases with high fever post‐treatment with sublingual misoprostol in Argentina (*n* = 6) and in Ecuador (*n* = 58) [18].

The overall incidence of high fever among women treated with misoprostol was (12.2% (6/49) [95% CI 4.6, 24.8]). Elevated temperature remained above 40.0°C degrees for less than one hour, and gradually declined over the next 3 h (Figure 2). The highest peak temperature recorded was 40.8°C. No cases of delirium were reported. Moderate and severe shivering, as described by the provider, were more frequent among women with high fever (83.3%; 5/6) than in women with mild/moderate fever (35.8%; 11/31; *P* = 0.030). Vital sign measurements also revealed a higher, though not statistically significant rate of tachycardia (pulse rate over 100 beats per minute) within the first hour post‐partum, among the high fever cohort (66.7%; 4/6), than in women without any fever (41.7%; 5/12; *P* = 0.317) or those with mild/moderator fever (54.8%; 17/31; *P* = 0.592). Other vital signs measured during the first hour post‐partum, including systolic and diastolic BP, did not differ significantly among the fever cohorts (data not shown).

Logistic regression analysis was performed to explore possible predictors to identify women at risk of developing fever after misoprostol administration. Because there were only six women with high fever in the study, we also assessed predictors of fever ≥ 38.0°C, which included 37 of 49 women treated with misoprostol. After controlling for various demographic and clinical factors, pre‐delivery anaemia (i.e. Hb < 11.0g/dl) was the only statistically significant predictor identified for the outcome of fever ≥ 38.0°C (OR 7.62 [95% CI 1.18, 49.0, *P* = 0.034]) (Table 3). Women who were anaemic before delivery had even higher odds of developing high fever (OR 18.3 [95% CI 0.78, 428, *P* = 0.071]), although this finding was not statistically significant (Table 3). The only two statistically significant predictors for high fever were a rapid placental expulsion and older age of the woman. Specifically, the odds of having high fever increased with each year of age (when included as continuous variable) (OR 1.29 [95% CI 1.10, 1.52, *P* = 0.001]). We also found that women who had a rapid placental delivery (i.e. within 5 min of birth) had increased odds of developing high fever, compared to those who delivered the placenta in > 5 min (OR 12.1 [95% CI 2.04, 72.3, *P* = 0.003]).

**Table 3:** Results from two multivariate logistic regressions to explore potential predictors of developing fever ≥ 38.0°C and high fever ≥ 40.0°C after treatment with sublingual misoprostol (800 mcg)

Factor	Fever ≥ 38.0°C		High fever ≥ 40.0°C	
	OR 95% CI	*P*‐value	OR 95% CI	*P*‐value
Woman’s age (continuous)	1.08 (0.94, 1.24)	0.284	1.29 (1.10, 1.52)	0.001
Gestational age (continuous)	1.54 (0.92, 2.59)	0.099	1.20 (0.59, 2.47)	0.615
Self‐ID’d ethnicity				
White	Ref		Ref	
Mestiza	1.17 (0.19, 7.17)	0.865	2.79 (0.36, 21.4)	0.325
Pre‐delivery Hb < 11.0g/dl				
No	Ref		Ref	
Yes	7.62 (1.18, 49.0)	0.032	18.3 (0.78, 427.7)	0.071
Labour induced/augmented				
No	Ref		Ref	
Yes	0.36 (0.25, 5.24)	0.458	0.33 (0.03 3.16)	0.334
Episiotomy performed				
No	Ref		Ref	
Yes	2.32 (0.36, 15.1)	0.377	7.73 (0.70 85.5)	0.095
Placental expulsion ≤ 5 min after birth of baby				
No	Ref		Ref	
Yes	2.23 (0.45, 11.0)	0.326	12.1 (2.04, 72.3)	0.006
PPH identified ≤ 15 min after birth of baby				
No	Ref		Ref	
Yes	1.61 (0.33,7.84)	0.557	24.4 (0.89, 673.5)	0.059

## Discussion

### Main findings

After treatment with sublingual misoprostol 800 mcg for PPH, temperature measurement confirmed that some women in Argentina experienced high fever (12.2% (6/49) [95% CI 4.6, 24.8]). This rate is significantly lower than the previously documented rate of high fever in Ecuador after treatment with the same misoprostol regimen (Argentina 12.2% *vs.* Ecuador 35.6% (58/163) [95% CI 28.3, 43.5], *P* = 0.002) [18]. None of the cases with fever in either study was life‐threatening, and the temperature patterns for fever (Figure 1) and high fever cases (Figure 2) confirm the transient nature of misoprostol’s thermoregulatory effects.

### Strengths and limitations

Results from this study respond to the call for continued vigilance of misoprostol’s adverse effects [12] and contribute important information on the safety of its use in Latin America, where misoprostol is widely available and used [29, 30]. Although oxytocin remains the drug of choice for PPH, there is growing evidence that prolonged oxytocin exposure in labour increases the risk of PPH and desensitises oxytocin receptors; which, in turn, reduces uterine contractility in response to oxytocin treatment [32, 33, 34]. It is plausible that reliance on misoprostol as a treatment alternative [3, 4, 6, 7, 8] may increase in settings that encounter oxytocin‐resistant PPH cases. Routine, systematic temperature assessment for two hours post‐treatment with misoprostol helps to clarify what providers and women should expect in terms of fever onset and duration.

This study has some limitations. First, the study’s sample size was small and did not include sites outside of Argentina. When planning this study, the procedures and data collection forms were carefully designed to enable a reliable comparison of high fever incidence with the previous study in Ecuador [18]. Ultimately, the sample of 49 women treated with misoprostol in this study was sufficient for the primary outcome analysis and revealed a significantly lower incidence than the study in Ecuador. However, there were too few cases of high fever to know for certain which factors might predict elevated temperature. The results in Table 3 should, therefore, be interpreted with caution and used only for hypothesis generation. A second study limitation is that the management practices of fever at the sites were not standardised. During the study training, management options were discussed with providers regarding what they could do to help alleviate symptoms of fever if needed, including applying cool compresses and giving acetaminophen. However, there is no evidence to suggest that these methods actually alter the trajectory of misoprostol‐induced fever [18, 19]. Lastly, these findings may have limited generalizability, especially if susceptibility to experience these side effects is due to genetic variants that are more common in some populations. Blood samples from women in this study will contribute to further genetic analyses, which will be reported on separately.

### Interpretation

Although the incidence of misoprostol‐induced high fever in Argentina is significantly lower than in Ecuador, both rates are still higher than high fever rates observed in other studies testing the same regimen conducted elsewhere [15, 16, 17, 18]. High fever rates from a multi‐country trial that systematically measured temperature at 60‐ and 90‐min post‐treatment with adjunct misoprostol (600 mcg sublingual) also warrant consideration [35]. Notably, at the site in Argentina, high fever was significantly more frequent than in the other four locations, including South Africa, Vietnam, Egypt and Thailand (Argentina: 14.5% (12/83); other sites: 5.8% (36/619), *P* = 0.003 (unpublished findings)). These trends, showing higher rates among South American populations, lend support to the explanation that genetic/population factors may play a role in misoprostol‐induced fever [23].

In the present study, results from logistic regressions did not confirm an association between women’s self‐reported ethnicity and misoprostol‐induced fever. Placental expulsion within 5 min of delivery was found to be significant predictor of high fever, a pattern that was identified in the previous study on misoprostol in Ecuador [15]. It was theorised that endogenous prostaglandins involved in placental expulsion [36], occurring together with exogenous prostaglandins (misoprostol) administered soon after delivery, increased the occurrence of shivering and fever in some women [18]. Pre‐delivery anaemia was also identified as a significant predictor of misoprostol‐induced fever. For various known reasons, anaemia can result in higher levels of pro‐inflammatory cytokine production [37, 38, 39] that, in turn, activates elevated prostaglandin expression [36, 40]. Applying insights from research on genetic susceptibility to misoprostol‐induced fever [23], it is plausible that genetic variants are responsible for some women having more expeditious prostaglandin transporters that resulted in heightened side effects.

Questions remain about who will develop high fever, but evidence shows that shivering and fever follow a predictable pattern [18, 22, 41]. A febrile response, triggered by misoprostol, is understood to occur when misoprostol crosses the blood–brain barrier and increases the thermoregulatory set point [18, 21, 23]. Shivering and increased heart rate are mechanisms that help to elevate the body’s temperature to this new set point [21]. In this study, nearly all women experienced both shivering and fever after treatment with misoprostol (83.8% (41/49)). Tachycardia was observed during the first hour post‐partum among four of the six women (66.7%) who developed high fever. Overall, monitoring for the onset of shivering after misoprostol administration may be the most practical way to know if fever should also be expected.

If body temperature is not measured, it is plausible that misoprostol‐induced fever may go unnoticed, especially if the woman’s bleeding is quickly controlled and monitoring of temperature does not extend beyond one hour. In the current study, nearly three‐quarters of women had their bleeding controlled with misoprostol alone within 30 min of taking the medication, at which time the average temperature was below 38.0°C. For most women, body temperature gradually increased post‐administration of misoprostol and did not peak until after 90 min, followed by a gradual decline.

Temperature assessment remains a core clinical parameter for identification of infection and is recommended for routine monitoring after delivery [42]. In obstetric early warning charts, a temperature over 38.0°C triggers a ‘red alert’ [31], which could result in the unnecessary administration of antibiotics. Thus, in clinical practice, knowing if and when misoprostol was given may assist providers in determining if the fever is an effect of the medicine or due to another reason [21, 43, 44].

## Conclusions

This study confirmed that the high fever rate at two Argentinian hospitals after treatment with misoprostol for PPH was significantly lower than the previously documented rate of high fever in Ecuador. Serious side effects from misoprostol are indeed rare, and the possibility for transient side effects, including high fever, should not deter use of this medicine to help control excessive bleeding from suspected uterine atony. Moreover, lower rates of high fever cases in the literature should provide reassurance that this effect is likely to be negligible in most settings. Overall, the clinical findings from this study contribute to a clearer understanding of misoprostol’s thermoregulatory effects, which may help providers and women know what to expect after sublingual administration of misoprostol 800 mcg for PPH.

## Data Availability

The dataset used for this analysis is available at Harvard Dataverse: https://doi.org/10.7910/DVN/GBKWCE
